# A Blockchain Protocol for Real-Time Application Migration on the Edge

**DOI:** 10.3390/s23094448

**Published:** 2023-05-02

**Authors:** Aleksandar Tošić, Jernej Vičič, Michael Burnard, Michael Mrissa

**Affiliations:** 1 InnoRenew CoE, Livade 6a, 6310 Izola, Slovenia; 2 Faculty of Mathematics, Natural Sciences and Information Technologies, University of Primorska, Glagoljaška 8, 6000 Koper, Slovenia; 3 Institute Andrej Marušič, University of Primorska, Muzejski Trg 2, 6000 Koper, Slovenia

**Keywords:** fault tolerance, blockchain, Internet of Things, edge computing, peer-to-peer, decentralized, sensor networks, verifiable delay functions

## Abstract

The Internet of Things (IoT) is experiencing widespread adoption across industry sectors ranging from supply chain management to smart cities, buildings, and health monitoring. However, most software architectures for the IoT deployment rely on centralized cloud computing infrastructures to provide storage and computing power, as cloud providers have high economic incentives to organize their infrastructure into clusters. Despite these incentives, there has been a recent shift from centralized to decentralized architectures that harness the potential of edge devices, reduce network latency, and lower infrastructure costs to support IoT applications. This shift has resulted in new edge computing architectures, but many still rely on centralized solutions for managing applications. A truly decentralized approach would offer interesting properties required for IoT use cases. In this paper, we introduce a decentralized architecture tailored for large-scale deployments of peer-to-peer IoT sensor networks and capable of run-time application migration. We propose a leader election consensus protocol for permissioned distributed networks that only requires one series of messages in order to commit to a change. The solution combines a blockchain consensus protocol using Verifiable Delay Functions (VDF) to achieve decentralized randomness, fault tolerance, transparency, and no single point of failure. We validate our solution by testing and analyzing the performance of our reference implementation. Our results show that nodes are able to reach consensus consistently, and the VDF proofs can be used as an entropy pool for decentralized randomness. We show that our system can perform autonomous real-time application migrations. Finally, we conclude that the implementation is scalable by testing it on 100 consensus nodes running 200 applications.

## 1. Introduction

Cloud computing solutions have driven the centralization of computing, process control (e.g., business information, manufacturing, distributed systems, IoT management), and data storage to data centers. Existing cloud-based solutions have few incentives, aside from reducing network latency, to redistribute their computing and storage resources. There are many reasons why centralization is more appealing. These range from legislative reasons, tax policies, and the availability and affordability of high speed internet and electrical power to reductions in maintenance costs and even climate preservation [1]. However, cloud computing solutions are struggling to address the specific challenges of emerging IoT and edge computing use cases.

**Figure 1:**
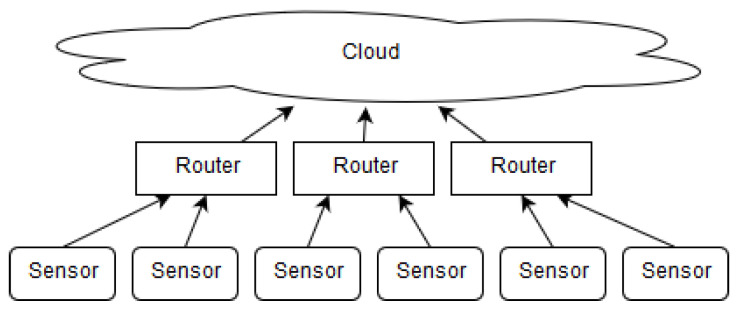
Standard sensor network architecture.

**Figure 2:**
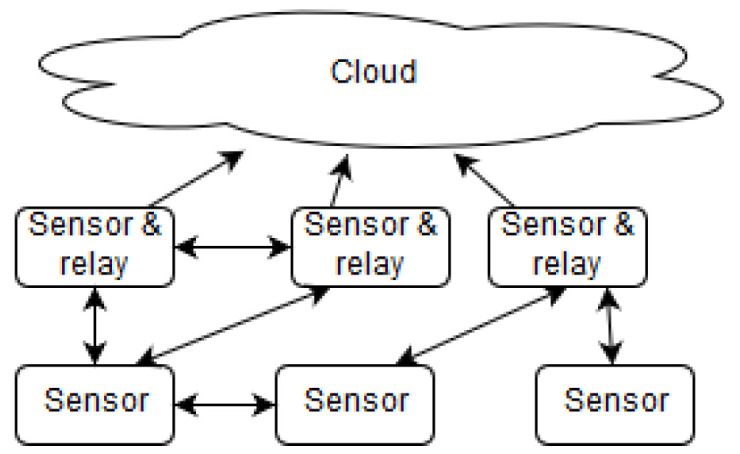
Mesh sensor network architecture.

The ever-growing number of devices on the edge causes scalability challenges for centralized architectures such as those based on cloud technologies. Edge devices tend to be heterogeneous, and existing IoT platforms remain isolated and unable to fully exploit their potential. Moreover, these devices have considerable computing resources, which, for the most part, remain underutilized, as most applications perform computation on the cloud. A major related challenge is supporting the homogeneous usage of edge devices, which requires applications to migrate at run-time from an overloaded device to a more available one. Currently, there is no standardized platform for general purpose computing that supports such run-time application migration. Another limitation to the large-scale deployment of sensor networks is the infrastructural investment needed to support the network, as typical architectures require a middle layer infrastructure that enables access to the cloud (Figure 1 and [2]).

We believe these challenges can be overcome, as recent technological advances have provided partial solutions and have presented new opportunities. These advances have paved the way for the recent paradigm shift from centralized to decentralized architectures for the IoT [3]. First, as edge devices are becoming more powerful and capable of running complex software, they provide a huge pool of available, yet underutilized, computing resources. Second, containerization solutions (as opposed to virtualization) have gained momentum and provide a pathway towards overcoming the heterogeneity problems while preserving acceptable performance [4]. Containerization software (e.g., Docker) provides software abstraction that enables general purpose computing on edge devices. Third, with the growth of edge devices capable of direct wireless communication, a mesh network approach has become worth exploring as a solution to reduce or eliminate the middle layer infrastructure needed for devices to connect to each other and to the cloud (Figure 2).

The need for edge computing is illustrated well by scenarios related to ad-hoc networks [5], and especially with peer-to-peer wireless sensor networks. The paradigm shift towards decentralization is relevant to numerous application domains such as smart building monitoring, structural health monitoring, self-driving vehicles, micro-service architectures, mobile devices, etc. In our work, we have experimented with a cultural heritage building located in Bled, Slovenia. We deployed several sensors to monitor the building’s state for maintenance purposes and its air quality to ensure safety for visitors. In cases where buildings are located in remote areas, as in our use case, edge devices must self-regulate and optimize their behavior at run-time. They must also have the capacity to scale up as the number of devices grows (scalability) and to adjust when dysfunctions occur, for example, when devices leave the network (experience byzantine behavior). In addition, the operation of all devices should be recorded safely for later analysis (transparency). In a cloud-based environment, edge devices send data to the cloud, where computation occurs. However, issues such as poor network coverage, frequent disconnection, the cost of infrastructural investment, inadequate dependability, and security concerns remain unaddressed [6,7,8]. Edge computing solutions attempt to reduce network latency; increase fault tolerance, dependability, and security; and reduce the cost of infrastructural investment needed to provide network coverage. They also operate independently of an external network connection. To address these issues, we propose an architecture based on an innovative combination of existing technologies. Specifically, our architecture provides a general purpose computation model allowing large scale sensor networks to distribute the computational load among edge devices (sensors, controllers, etc.). The solution uses containerization so that applications can be built using any programming language or stack and to provide an abstraction layer between the application requirements and the host hardware. The decision-making process for resource allocation is carried out by a decentralized orchestrator implemented as a consensus protocol that outputs a migration strategy, which is in turn stored on the blockchain (a blockchain is a growing list of records called blocks, linked together using cryptography so that the contents cannot be modified without breaking the list, and the nodes follow a shared consensus protocol to validate new blocks). It features high fault tolerance, full transparency, reduced network infrastructure cost, and no single point of failure. The network layer uses decentralized randomness to constantly change the network topology to allow efficient propagation of information pertaining to the resource utilization of nodes.

The main contributions in this paper are as follows:

- Introducing a decentralized architecture capable of run-time application migration for large scale deployments of peer-to-peer IoT sensor networks;
- Describing three key contributions:
  – A scalable consensus protocol layer;
  – An efficient, secure, and dynamic topology based on k-means clustering;
  – A decentralized orchestrator capable of low-latency, real-time application migrations;
- Evaluating each contribution by performing empirical tests with a reference implementation of the protocol;
- Improving migration times by implementing CRIU, an experimental feature of Docker that allows the system to migrate an application’s state without affecting its run-time;
- Showing that distributed consensus and application management is possible at run-time, opening the door to several improvements towards self-managing IoT platforms;
- Demonstrating that blockchain overhead is a negligible aspect of the actual cost of application migration and that the system is able to finalize blocks with slot times as low as 5 s while maintaining higher decentralization than existing platforms;
- Identifying network instability (devices entering and leaving the network) as a potential area for future exploration and proposing solutions to reduce the required computational power while maintaining optimal application management;
- Extending the algorithm governing the decentralized orchestrator to allow applications to submit migration policies that the orchestrator will have to follow during its operation.

The rest of this paper is structured as follows: Section 2 provides the necessary background knowledge and overviews the most relevant related works to highlight the originality of our proposal. Section 3 details our architecture and its operation. Section 4 describes our evaluation environment Section 6 summarizes the results and gives guidelines for future work.

## 2. Background Knowledge and Related Work

The most critical unmet challenges in monitoring edge devices according to [9] are: mobility management, scalability and resource availability at the edge of the network, coordinated decentralization, interoperability and avoiding vendor lock-in, optimal resource scheduling among edge nodes, and fault tolerance. No widely used cloud-based monitoring tool for edge computing fully addresses these challenges. Some requirements remain unmet when using any existing solution, as many system aspects, including containerization and end-to-end network quality, are not adequately addressed [9]. The EU project RECAP [10] presents a vision of the next generation of intelligent, self-managed, and self-remediated cloud computing systems (i.e., a system that can monitor and relocate resources to achieve Quality of Service—QoS). The project also describes models intended to be integrated in network topology-aware application orchestration and resource management systems from an edge computing perspective [11]. Another solution, AutoMigrate [12], incorporates a selection algorithm to determine which services should be migrated to optimize availability. Although this system has solutions for most of the problems we address, it does not resolve the single point of failure (SPOF) issue because it relies on a central service to orchestrate migrations. Our decentralized implementation eliminates the SPOF issue.

### 2.1. Orchestration Solutions for Edge Computing

Orchestration denotes control by a single entity over many. This differs from choreography, which is more collaborative and allows each involved party to describe its part in the interaction [13]. We have identified the most successful orchestration solutions to be Kubernetes [14], which is the most used and most feature-rich orchestration tool [15]; Docker Swarm (https://github.com/docker/swarm accessed on: 25 April 2023); Amazon Web Service Elastic Container Service [16]; the Distributed Cloud Operating System (https://dcos.io/); and Nomad (https://www.hashicorp.com/products/nomad accessed on: 25 April 2023).

The Decenter EU project (Decenter project homepage: https://www.decenter-project.eu) proposes decentralized orchestration technologies for fog-to-edge computing. Although the project does support decentralized orchestration between multiple domains and records service-level agreements and violations to the blockchain, the solution is designed as a federated approach where a multi-domain orchestrator maintains several domains that, in turn, are driven from local orchestrators [17], thus still showing an SPOF with the multi-domain and local orchestrators. The project also implements a blockchain to act as a brokerage platform where smart contracts guarantee resource sharing across domains [18]. In contrast to a federated approach, our implementation is fully decentralized, with a randomly selected orchestrator at each interval, thus avoiding the SPOF problem and not relying on a trusted third party.

All of the architectures discussed above have a common flaw: the SPOF problem. In each case, the flaw is characterized by a single orchestration entity. Most solutions also lack support for edge devices. Our proposed solution addresses these shortcomings, while providing full transparency, variability of the system, and a completely decentralized operation backed by a strongly secure, scalable, and efficient consensus mechanism.

Recently, a decentralized protocol for the orchestration of containers named Caravela was proposed [19]. The solution relies on Chord for resource discovery and employs a volunteer system in which nodes are categorized as suppliers (supplying resources), buyers (searching for resources), and traders (mediating supply/search for offers). The authors show that their solution can scale using a random migration algorithm, but fails fulfill deployment requests. It is also not able to fulfill the global binpack scheduling policy due to a lack of global shared states.

Our proposed protocol extends the previous state-of-the-art Caravela [19] in the following ways:

- Caravela uses a market-based model in which edge nodes require a reward in the form of digital currency for the network to operate. Caravela proposes using Bitcoin but considers its implementation out of scope. Our solution does not require a market model and an external digital currency as nodes also maintain a blockchain network.
- Caravela performs resource allocation in a more sophisticated way, offering multiple heuristics such as binpack and round robin. However, the orchestrator is not able to migrate applications at run-time. Container resource consumption is dynamic, and the system must adapt to changes. Our protocol performs both initial scheduling and run-time migrations.
- Caravela is completely decentralized, where actors offer resources to buyers. It is more scalable, as it does not maintain a global log of all containers. Our protocol is able to achieve comparable results and maintain a blockchain as an immutable, verifiable, and secure log of the entire network state.

A similar light-weight, blockchain-enabled architecture was proposed by Cheikhrouhou et al. [20], which uses a round-robin-based block proposal consensus. However, the proposed protocol does not guarantee liveliness in a permissionless setting. Nodes propose blocks in a predictable round-robin algorithm based on their unique identifiers, which in turn opens up the consensus layer to denial of service (DOS) and collusion attack vectors.

Similarly, Cheikhrouhou et al. [21] propose a proof of trust based consensus mechanism, with the aim of securing message exchange in EV environments powered by the IoT. However, their consensus mechanism assumes a private network in which nodes do not exhibit Byzantine behavior. Containers, as used in this paper, are a group of namespaced processes running within an operating system. Docker is the most widely used container solution [22] and one of the few platforms that can migrate apps at run-time and enable easy communication. For these reasons it was used as the main testing platform.

### 2.2. Available Blockchain Solutions

Ever since the inception of the Bitcoin protocol, blockchain protocols have been extensively researched, and blockchain-based solutions apply to many domains, as shown in [21]. Most notable advances contribute to improving the consensus mechanisms that would address the notorious energy inefficiency of Bitcoin’s PoW consensus. In a PoW-based consensus mechanism, nodes (miners) compete for the right to append new blocks to the chain by computing a solution to a difficult problem. Appending new valid blocks is rewarded with digital assets, which encourages miners to contribute more computational resources in order to increase their rewards. The protocol periodically adjusts the difficulty of the problem to meet the target block time of approximately 10 min.

To address the inefficiency of PoW, new consensus mechanisms have been proposed, the most notable ones being proof of stake (PoS), proof of authority (PoA), Tendermint, and proof of history (PoH).

Proof of stake greatly reduces the energy footprint over PoW by selecting block-producing nodes based on their stake in the digital asset the blockchain maintains. To achieve this, a source of entropy for decentralized and secure randomness is needed. Nodes are selected at random, normalized with their stake. In public networks, the consensus mechanisms make the assumption that nodes participating in the consensus are financially incentivized against Byzantine behavior to avoid risk of financial loss. To achieve this, penalties for nodes violating the protocol are put in place [23]. Many consensus mechanisms are based on PoS, with various trade-offs between security, decentralization, and scalability. However, on a technical level, they can be categorized as vote-based consensus mechanisms in which a subset of nodes is chosen to vote (attest) for a proposed block. If the proposed block gathers sufficient votes, it is considered accepted and is thereby added to the chain. The choice between aforementioned trade-offs stem from the basic CAP theorem [24]. The subset of nodes participating in the consensus at each round (block) is significantly smaller then the set of validators (consensus nodes). While this decreases decentralization, it greatly improves scalability, as gathering and aggregating a large number of votes is not feasible. However, this stresses the importance of unpredictability that arises from a secure source of randomness [25]. In PoW-based blockchains, unpredictability comes from the mining process. Obtaining the proof requires randomized guessing (unpredictability), which is provably fair and uniform.

For PoS consensus mechanisms, many sources of randomness have been proposed. Most notable are RANDAO [26], verifiable random functions [27], and, recently, verifiable delay functions [28].

The proposed solution makes use of a blockchain to store the state transitions of the network in a verifiable and transparent way. Unlike existing blockchains, which either use an account-based model [29] or a UTXO model [30], our blocks do not store transactions or account states. The block structure is tailored to accommodate application migration and the verifiability of migrations. Hence, the blocks are snapshots of the state of the system containing information about the available resources and the required resources of applications managed by the system.

A survey of the most notable, readily available blockchain solutions for private networks yielded three candidates:

- Implementing a private Ethereum network, although the implementation is fairly simple [31]. The available consensus mechanisms include PoW, which is not secure for networks with no value, and proof of authority (PoA), which limits the consensus nodes to a subset of trusted nodes, thereby decreasing decentralization and security.
- Implementing a HyperLedger blockchain in all configurations requires notable CPU burdens [32]. As the number of nodes in the network grows, the system requirements scale far beyond what can be considered sustainable for edge devices.
- MultiChain (MultiChain Open source blockchain platform: https://www.multichain.com/) also presents a viable alternative for a private blockchain network [33], but it is also not suitable for edge devices [33]. Moreover, it is primarily focused on facilitating cryptocurrency and asset transactions.
- Solana [34] uses verifiable delay functions as a source of entropy for their leader rotation algorithm. However, their VDF implementation requires thousands of graphical processing units to meet the speed requirements, which is not suitable for edge devices.

All the presented available off-the-shelf solutions satisfy most of the criteria posed by the research question, but they all rely heavily on computational power, which makes them unsuitable for edge devices. Furthermore, the required block structure and changes on the protocol would outweigh the benefits and accumulate technical debt.

### 2.3. Decentralized Self-Managing IoT Architectures

A survey of the scientific literature shows multiple solutions that address decentralized self-managing architectures for the Internet of Things (IoT). The most notable examples are as follows:

- Maior et al. [35] present a theoretical description of a decentralized solution for energy management in IoT architectures. The solution is aimed at smart power grids. They present four algorithms with analyses of correctness in order to describe the behavior of self-governing objects.
- Higgins et al. [36] propose a distributed IoT approach for electrical power demand management.
- Suzdalenko and Galkin [37] extend the approach by Higgins et al. [36] by allowing users to individually join and depart the environment at run-time.
- Niyato et al. [38] propose a system that addresses home energy management where devices communicate directly among themselves.
- dSUMO [39] address the synchronization bottleneck by proposing a distributed and decentralized microscopic simulation (the focus is on data throughput and not fault tolerance; throughput is increased using a decentralized setting).
- Al-Madani et al. [40] address indoor localization utilizing Wireless Sensor Networks (WSNs) and relying on a publish/subscribe messaging model. The results show that Really Simple Syndication (RSS) [41] achieves acceptable accuracy for multiple types of applications.
- Auyla et al. [42] present a blockchain-based, multi-layered, edge-enabled, secure data processing framework for an edge-envisioned vehicle-to-everything environment. The solution is evaluated in terms of its latency, energy consumption, and SLA agreement compliance.
- Singh et al. [43] propose a secure architecture based on a one-time signature scheme for the IoT in an edge infrastructure. The solution relies on a blockchain-enabled distributed network. The study shows favorable results compared to available solutions in terms of computing time and communication cost.
- Kanagachalam et al. [44] present BloSIM, which is a blockchain-based service migration for connected cars in an embedded edge environment. They deployed edge clusters using NVIDIA Jetson boards in an embedded edge environment. Kubernetes was used as a container orchestration instead of a decentralized consensus protocol. The authors used similar methodology for the evaluation through performance parameters such as latency, throughput, storage, and bandwidth.

Our proposed solution differs from the previous contributions in two ways:

- We consider multiple criteria to optimize and include a framework to add more criteria in the future. Other solutions typically focus on a single problem and presenting an optimal solution for it. We argue that an IoT architecture requires multiple optimization criteria.
- Our protocol is highly decentralized, as it allows all nodes to participate in the consensus while maintaining low hardware requirements fit for edge devices.

A related approach by Samaniego and Deters [45] suggests using virtual resources in combination with a permission-based blockchain for provisioning IoT services on edge hosts. They use blockchain to manage permissions only and therefore provide security using blockchain. In contrast, our approach uses blockchain to store all information about service choreography, which makes it verifiable over time, while still providing security.

The main contribution of this paper is a light-weight blockchain protocol that can achieve high decentralization and has low hardware requirements, making it suitable for the hardware typically found in edge devices. The proposed protocol inherits ideas from Ethereum 2.0 but replaces the source of entropy needed for consensus with a VDF. Moreover, the structure of the block carries the state transition information, and unlike existing blockchains, it does not have the concepts of accounts, balances, and transactions. The acronyms used in this paper are presented in Table 1.

## 3. Proposed Decentralized Architecture

In this section, we provide a general description of our architecture [46] and highlight its main components. The main purpose of our architecture is to enable the verifiable and decentralized management of applications on the edge. In our vision, applications can be built as containers and submitted to the network by reaching any node via an API. We use containerization to decouple the host running the application from the application itself and address the issue of hardware and software heterogeneity. This allows the protocol to assume an application can be run on all nodes in the network. A randomly selected and decentralized orchestrator on the network would then be able to choreograph the execution of the application and migrate applications between hosts at run-time. As our architecture is fully decentralized, each node is locally driven by a protocol that participates in establishing the global state of the network via a specially built consensus mechanism. Nodes in the network reach consensus on a migration plan in an effort to improve the resource allocation of running applications. A migration plan is viewed as a state transition, which is stored on the blockchain formed by the participating nodes. We implement a choreographed solution, which is a collaborative, rather than a directed, approach (such as orchestration). Choreographed systems define a way for each member to describe its role in the interaction [13]. This collaborative approach avoids the SPOF problem. Despite this advantage, there are no choreography solutions known to address the open problems described at the start of the Section 2.

To provide a global understanding of our fully decentralized architecture, we first describe the architecture of a single node, followed by the interaction protocols between nodes.

All identified orchestration solutions presented in Section 2.1 rely on a primary/replica model. The main service selects the applications that need to be reallocated according to a selected optimization algorithm.

Our migration algorithm is able to:

- Pause a container;
- Transfer the context to a different host;
- Resume the execution given the context.

Additionally, we implemented migrations using checkpoint/restore in userspace, or CRIU, an experimental feature available in Docker [47].

### 3.1. Overview of Node Architecture

**Figure 3:**
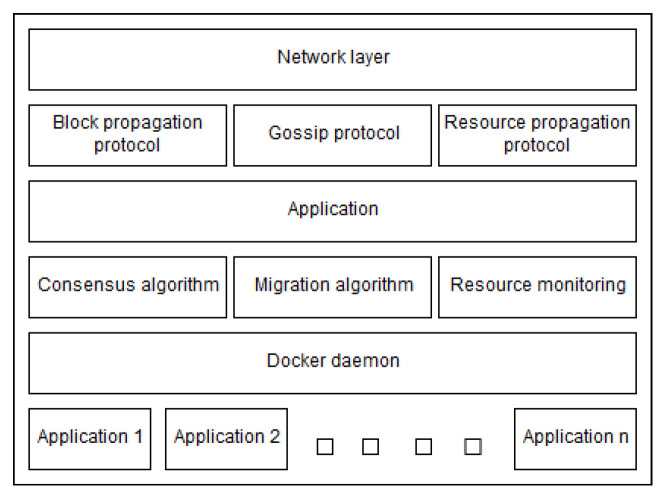
General overview of a node architecture.

The node application that we developed is containerized in Docker. As shown in Figure 3, the internal architecture of a node is composed of the following modules that support application management:

- Networking layer: this layer deals with network communication through deployed APIs;
- Gossip protocol: A rendezvous-based gossip protocol is used to build a distributed hash table (DHT) that maps public IP’s of nodes to their network address. The messages are encoded using protocol buffers (https://developers.google.com/protocol-buffers accessed on: 25 April 2023), which is the underlying protocol that makes sure all messages reach all nodes in the network while minimizing network usage;
- Block propagation protocol: relies on the gossip protocol to spread newly accepted blocks over the network;
- Resource propagation protocol: relies on the network layer to deliver the state of resources (currently CPU, RAM, disk usage, and network utilization of Docker containers) over the network to the receiving node;
- Consensus protocol: ensures all nodes reach consensus in a decentralized way (presented below);
- Migration algorithm: guarantees that a migration strategy is reached whenever needed thanks to a deterministic algorithm. This algorithm is executed at each slot until the proposed block is accepted and finalized. The output of the algorithm is included in the block to construct a verifiable and transparent log of each application’s life-cycle;
- Docker daemon: hosts applications and is used for abstracting the underlying heterogeneity between devices, systems, and applications. It provides support to our solution via Docker APIs;
- Resource monitoring: relies on the Docker API to monitor the state and resource allocations of the hosting device and the applications running on it.

### 3.2. Storing System States in a Blockchain

All nodes share information about their states through a federated type topology obtained by distributed clustering of nodes, which is explained in more detail in Section 3.3. We define a state as a matrix of vectors describing the resource consumption associated with each application. A *resource pool* data structure is replicated in all nodes and contains information about all node states. In our use case, we define a vector with the following values:

{app,cpu,ram,disk,network,timestamp}

This provides a time series of system resource utilization for each application across an operating period. The resources used by applications are obtained through the Docker API and represented in percentages for simplicity. Taken at a specific time interval, the vector is a block that includes a list of per-application resource statistics, as shown in Table 2, where Nodeis a 256-bit hash representing the system-wide unique ID of the application, RAM, DISK, and CPU are floats representing the portion of node’s available resources used by the application. Finally, the average latency is computed as the 30 s moving average of round-trip delay (RTT) towards randomly selected validators.

From a data block, it is then possible to compute a migration plan to optimize the allocation of applications to nodes according to the resource states of all nodes. The migration plan is also included in the block, which produces a transparent computational log for verifying if the adopted migration plan was actually efficient and fair. The architecture does not enforce any specific migration algorithm. The only constraints are that the algorithm must be deterministic and must rely only on data included in the block (reached by consensus). For the same inputs to a deterministic algorithm, proposed migrations can be verified much like transactions are verified in public blockchains: using basic asymmetric cryptography.

**Figure 4:**
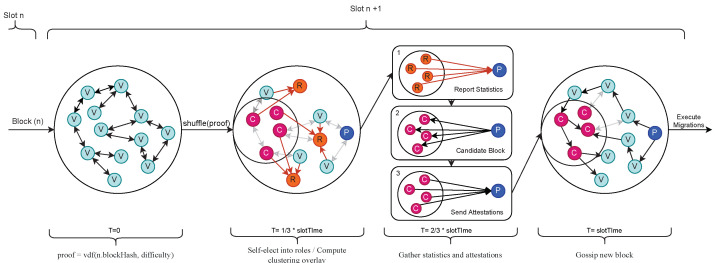
Diagram of the time-synchronous protocol depicting the important roles and actions each node executes for each slot.

In order to provide liveliness and responsiveness, delivering resource consumption statistics to the block producer (P in Figure 4) must be faster than $\frac{2}{3}\cdot slotTime$. Using the gossip protocol produces unwanted latency, and greatly increases resource utilization when maintaining the message queue (MQ). To overcome this, we implement a distributed k-means clustering algorithm that requires no communication between nodes to compute. Clustering is used to group nodes in a separate overlay network where statistics are propagated using the UDP protocol. The seed used to compute k-means is shared by all nodes, the VDF proof. Cluster representatives (R in Figure 4) are nodes that are responsible for requesting resource utilization statistics from their members, and transmitting them to the block producer. The timing details are strongly intertwined with the synchronicity of the consensus algorithm further explained in Section 3.4.

This federated overlay topology greatly decreases decentralization and, consequently, fault tolerance in case a cluster representative exhibits Byzantine behavior. However, this is of limited concern considering that a failure to disseminate resource utilization only delays potential migrations for a subset of applications in the current block. Once a new block is accepted, a new overlay topology is computed. Eventually, a node exhibiting Byzantine behavior will be excluded from the validator set as, detailed in Section 3.4.

### 3.3. Migration Algorithm and Verifiability

To forge a block, nodes compute a migration plan based on resource statistics in the previous block. The migration plan is executed once the proposed block is accepted. Application migration is realized using Docker commands to pause the application, compress it, and transfer it to the destination node where it is restored. Preferably, CRIU is used and only the state of the running container is extracted and migrated. All migration plans are securely stored in the blockchain for eventual verification. The time to produce a block is configurable and largely depends on the requirements for responsiveness, resource availability, and network size. However, there are some lower bounds set by the consensus protocol (empirically, 5 s) under which we experienced occasional block and vote propagation issues, as well as aggregation delays that can cause unplanned soft forks.

Each block contains data that describe the states of nodes and the migration plan resulting from the application of the generation algorithm. Each block also contains the signature of the previous block to follow the principles of the blockchain, so that all blocks are dependent on their previous blocks, which makes it irreversible. To demonstrate our approach, we relied on the Algorithm 1 to generate migration plans according to the resource pool. Blocks also include meta-data that facilitate their utilization, such as the block’s hash, the previous block’s hash, VDF proof, aggregated votes, validator set updates, slot, and epoch.
**Algorithm 1** Deterministic migration plan generation.**Input:** BlockData**Output:** Generation plan  Max⇐FindMaxLoadedNode(BlockData)  Min⇐FindMinLoadedNode(BlockData)  **if** !AppQueue.isEmpty() **then**     **while** !AppQueue.isEmpty() **do**       Min⇐FindMinLoadedNode(BlockData)       Min.addApp(AppQueue.dequeue())     **end while**   **else**     AppToMigrate⇐Max.MaxLoadApp     DeltaScore⇐(Max.score−Min.score)     NextDeltaScore⇐(Max.score−AppToMigrate.score)−(Min.score+AppToMigrate.score)  **end if**  **if** Math.abs(DeltaScore>NextDeltaScore) **then**     Migrate(AppToMigrate, Min)  **end if**

### 3.4. Consensus Mechanism

A key component of a blockchain is the ability for nodes to reach consensus on the global state of the ledger. With increasing interest in blockchain technology in recent years, many consensus algorithms have built upon basic proof of work (PoW) [48] concepts. PoW is a technique that protects from various attacks by requiring a certain amount of processing power to use a service, which makes a potential attack worthless because it becomes too costly. However, most algorithms used in permission-less blockchain implementations rely on basic game theory assumptions, which hold only when the blockchain facilitates value transfers and actors can be assumed to behave in their own (financial) interests (i.e., the nothing at stake problem).

In permissioned networks, where there is usually no monetary value, the consensus algorithms used in monetary blockchain implementations are not appropriate. Instead, a known family of consensus algorithms for permissioned networks can be used based on voting schemes for leader elections such as Practical Byzantine Fault Tolerance (PBFT) [49], Proof of Elapsed Time (PoET) [50], or RAFT [51]. However, these algorithms require multiple messages to be sent through the network in order to commit a change, thereby increasing network utilization and delay.

Our algorithm is based on a random draw that is universally verifiable. To achieve decentralized randomness and verifiability, we make use of Verifiable Delay Functions (VDF) [28]. A VDF is a function that takes a large, but configurable, quantity of non-parallel work to compute and produces a verifiable proof. More specifically, VDFs are similar to time lock puzzles but may require a trusted setup where the verifier prepares each puzzle using its private key. Additionally, a difficulty parameter can be adjusted to increase the amount of sequential work, thereby increasing the delay. A VDF can be decomposed into three main phases, namely:

1. **A setup** phase, where a security parameter ($block_{hash}$) and a difficulty parameter *d* are given;
2. **An evaluation** phase, which computes the VDF from the given input to produce a proof *p*, which is meant to be infeasible to compute in less then time(d);
3. **A verify** phase, which is a deterministic algorithm that outputs either *true* or *false* on input $p,block_{hash},d$. Verification should be much faster than evaluation.

A VDF construction must meet the following properties:

1. **Correctness**, which requires that the output to an honest evaluation always outputs true in **Verify**;
2. **Soundness**, which ensures that obtaining a positive output from **Eval** given a malicious output is negligible;
3. **Sequential**, which guarantees honest parties can compute a VDF in *t* sequential steps and no adversary can compute it in less than *t* with parallelization.

We extend our previous consensus algorithm [46] such that nodes first compute a VDF depending on the difficulty assigned for block n+1 and desired *slotTime*, which is configurable and allows the operator to set the desired *slotTime* directly effecting network performance. We then use the proof $P_{n}=VDF\left({\left(n-1\right)}_{hash},{\left(n-1\right)}_{difficulty}\right)$ as a decentralized entropy pool for a random number generator (RNG) to draw decentralized randomness for a given *slot*. Figure 4 illustrates the time-synchronous protocol executed for each slot. Nodes are able to self-elect into consensus roles (e.g., *Block Producer (P)*, *Validator (V)*, *Committee Member (C)*), as outlined in Algorithm 2. Due to the seeded RNG, all nodes compute the same assignment of roles for all participating nodes, thereby not requiring any message exchange to agree on their roles. Moreover, the canonical nature of the chain provides some security guarantees so that the roles for the future block n+1 cannot be computed before block *n* is accepted. Once roles are assigned for a given slot, nodes perform their sub-protocols as follows:

1. The *Block Producer (P)* is a singular node elected to each slot to produce a candidate block. The candidate block is sent to all committee members. Upon sending, the block producer listens for attestations for $\frac{2}{3}*slotTime$ and aggregates them. The aggregated signature is then included in the block header and gossiped to the entire network if a sufficient number of votes are received; otherwise, a skip block is proposed.
2. The *Committee Members (C)* are responsible for attesting to candidate blocks. They verify the block integrity, signatures, and data to produce a Boneh–Lynn–Shacham signature (BLS), then send the signature to the block producer.
3. *Validator (V)* nodes receive a new block and verify the integrity and committee signatures to decide to either accept or reject the block.

The protocol assumes all validating nodes form a validator set, which is shared among all nodes participating in the consensus protocol. The assumption is guaranteed by logging inclusions and exclusions in blocks. To build the validator set, a node builds the chain to the current tip (last block), and upon verifying each block, it executes the validator state transition function to reconstruct the validator set. The state transaction function simply stores changes to the membership of the validator set. Nodes that want to participate in the consensus gossip their signed inclusion request, and once included into a block, they are considered in the validator set by all nodes simultaneously and can begin participating by role self-election. Nodes are excluded from the validator set when they are elected to the role of *Block Producer (P)* and then fail to deliver the candidate block to the committee in time. The committee will then vote for a skip block, which includes only the exclusion of the *Block Producer (P)*. In a permissioned setting, this is considered sufficient to evaluate future failures in case a node is faulty. The node can rejoin the validator set at any time by gossiping an inclusion request. We define the consensus protocol more formally in Algorithm 3.
**Algorithm 2** Role election.**Input:** Slot, ValidatorSet**Output:** Roles[]  
$Slot_{seed}⇐VDF\left(chain_{\left(slot-1\right)}.hash,chain_{\left(slot-1\right)}.difficulty\right)$  
$ValidatorSet⇐Shuffle(ValidatorSet,Slot_{seed})$  
$Roles{[}^{\prime }blockProducer^{\prime }]⇐ValidatorSet.subset\left(0,1\right)$  
$Roles{[}^{\prime }committee^{\prime }]⇐ValidatorSet.subset\left(1,committeeSize\right)$  
$Roles{[}^{\prime }validator^{\prime }]⇐ValidatorSet.subset\left(committeeSize,ValidatorSet.size\right)$

**Algorithm 3** Consensus**Input:** Role[]**Output:** ∅  **switch** (Roles[nodeId])  **case** blockProducer**:**    
block.migrations⇐prepareMigrationPlan(containerStats)    
block.signature⇐sign(block)    
broadcast(block,committee)    
$votes⇐await(\frac{slotTime}{3})$    
block.votes⇐BLS.aggregate(votes)     **if** 
hasMajority(block.votes) 
**then**          gossip(block)     **else**          skipBlock()     **end if**  **case** committee**:**     $candidateBlock⇐await(\frac{slotTime*2}{3})$     **if** candidateBlock==null **then**         skipBlock()     **else**         proof⇐verify(candidateBlock.proof)         migrations⇐verify(candidateBlock.migrationPlan)         signature⇐verify(candidateBlock.signature)         **if** (proof & migrations & signature) **then**             send(vote,blockProducer)         **else**           skipBlock()         **end if**       **end if**    **case** validator**:**       block⇐await(slotTime)       **if** block==null **then**         skipBlock()       **else**         proof⇐verify(block.proof)         migrations⇐verify(block.migrationPlan)         signature⇐verify(block.signature)         votes⇐verify(block.votes)         **if** (proof & migrations & signature)& votes) **then**           chain⇐block         **end if**       **end if**    **end switch**

### 3.5. Security and Fault Tolerance Considerations

Fault tolerance is an important property of the system which must guarantee the liveliness of applications running at any given time. Hence, the risk of accidental forks (a split in the blockchain) is a concern. In a permissioned setting, forks are accidental and are a product of node failures or message propagation delays. We provide various scenarios of forks below and show how the fork choice rule addresses them:

1. The proposed block *b* for the current slot *s* is not propagated to all committee members in time *C*. A forked subset $C_{f}\subset C$ votes and includes a skip block sb for slot *s*:
  (i) When $\frac{\left|C\right|}{2}>C_{f}$, block *b* will pass the majority vote, and the tip of the chain is *b*. However, $C_{f}$ tip is sb. In this case, $C_{f}$ will produce different role assignments and attempt to build on sb. Even if the block producer $bp\in C_{f}$, a majority vote cannot pass as $\frac{\left|C\right|}{2}>C_{f}$. Therefore, $C_{f}$ will add another sb. Eventually, the real block will reach the forked nodes, and due to a hash mismatch, nodes will initiate the fork resolution protocol.
  (ii) When $\frac{\left|C\right|}{2}<C_{f}$, block *b* will **not** pass the majority vote, and the tip of the chain is sb. No fork will occur.
2. Alternatively, attestations for *b* can be aggregated in time, but *b* fails to propagate to all committee members in time. A subset of committee members may then assume the block producer experienced a fault and start gossiping sb. A network partition in the validator set occurs due to a race condition. However, eventually *b* will reach nodes with the tip sb. Due to a hash mismatch, they will initiate the fork resolution protocol.

**Figure 5:**
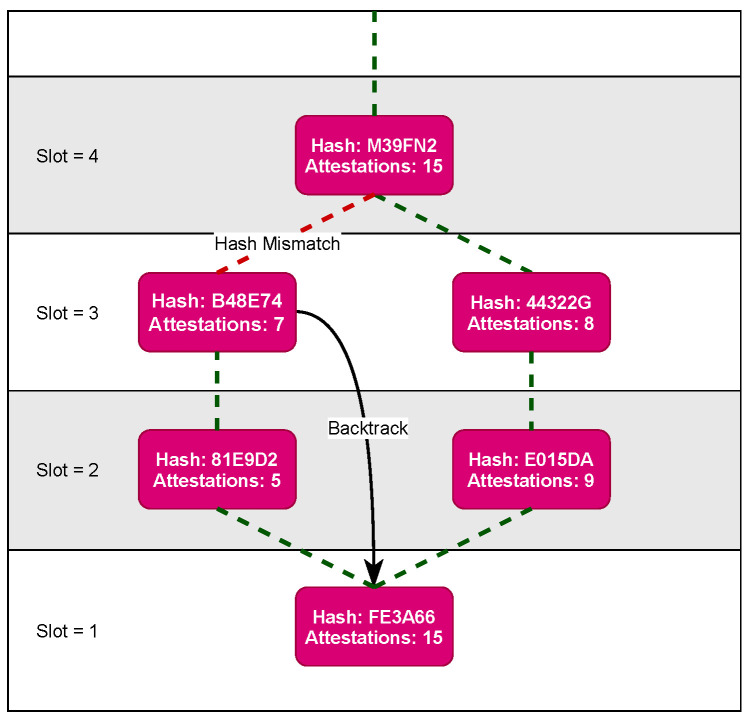
Fork resolution protocol.

Figure 5 illustrates how fork resolution works. At height 2, two different blocks are proposed and accepted. Both reference the correct previous block hash in which all nodes agreed on the same previous block n+1. However, any blocks after height 2 will have a different previous block hash. Eventually, one of the chains has to be dismissed. For each of the aforementioned cases where a fork can occur, this eventually happens. In case of disconnect or high latency, the network eventually reaches higher connectivity as peers build new connections. Moreover, for each slot, nodes take up new roles in the consensus protocol, and the likelihood of effected nodes to maintain the same roles decreases rapidly.

In Nakamoto-style [30] consensus algorithms, the fork choice rule states that the longest chain (most proof of work) is the correct chain. However, a vote/role-based consensus reduces variance in block time and the forked chain can have an identical block height. Instead, when a node cannot add a new valid block due to a mismatch with the previous block hashes, it backtracks to recheck the attestations for each block down to the forked block. Afterwards, it rebuilds the chain including the blocks by following the chain with most cumulative attestations. Note that in order for a node to receive a valid block with a different previous block hash, the network partitions/high latency must have been resolved for the node to receive the alternative chain.

Another aspect of forks is the fault tolerance related to applications running in the system. Every block includes a migration plan, and in case of a fork, two or more migration plans are created and accepted by two disjoint sets of nodes. A migration plan will include all applications, which guarantees the liveliness of and variability in computation. Moreover, in case of a chain split, both sets of nodes (*A*, *B*) will execute the their respective plans, which can unfold in the following two ways:

1. An application λ is planned to migrate from a node in *A* to a node in set *B*, or vice versa;
2. An application λ is planned to migrate to another node within sets *A* or *B*;
3. An application λ does not need to migrate in either chain.

In order for an application to migrate from one node to another, a direct connection between the nodes must be established, where one node sends a compressed version of the container to the other. To execute this, both the origin and destination node must agree and run the migration protocol. If a fork occurs due to high network latency or complete disconnection between the two sets, the migration protocol will attempt to communicate between the sets, resolving the fork as shown in Figure 5 as long as communication is possible. Whenever a migration plan requires an application to migrate between two conflicting chains, it forces a fork resolution. Moreover, in such cases, only one migration is run at the same time. However, in the event application λ is planned to migrate within its originating chain, the migration will not force the network to reconnect. This could be considered a hard fork, as there is no connection between the two networks, and results in separate instances of the network. The final example is when application λ is not required to migrate, in which case one instance remains running and the system is not affected. The only example where serious faults might occur is when the network is not well connected and forks occur when one partition accepts a block, while the rest vote for a skip block. However, the migration algorithm is deterministic and the input is in the previous block. This means both block producers will produce the same migration plan even though the block hashes will be different. Although there will be two conflicting chains, the migration plan will be the same. Once the partition is solved, the forked chain with a skipped block will revert back to the correct chain.

## 4. Evaluation and Empirical Results

To assess the performance of our implementation, we designated a specific node to record operational information about the entire system in a time series database. Our experimental environment utilized Docker Swarm technology to establish a cluster of eight nodes, each featuring a 16-core (32-thread) Ryzen Threadripper CPU and 32 GB of RAM. The nodes within the cluster were interconnected by an overlay network with negligible latency. To simulate more realistic network conditions, we introduced artificial latency on individual UDP packets in the form of a random delay within the range of 0–5 milliseconds.

The Docker service deployed a containerized node software across the cluster, which balanced the load across nodes. This network was designed as a Docker service, which commenced a new node every 10 s to avoid congested network conditions. The bootstrap setup was established with each test-net, where the first node was designated as the bootstrapping node, with its public key and IP address known to all other nodes.

Each node was restricted to one CPU core and 256 MB of RAM, exceeding the protocol’s requirements. Furthermore, the test applications were implemented as Docker images, which were executed through a Docker daemon within each node’s Docker container instance. This two-tiered abstraction facilitated the separation of node resources from application resources, permitting the test-net to operate seamlessly.

**Figure 6:**
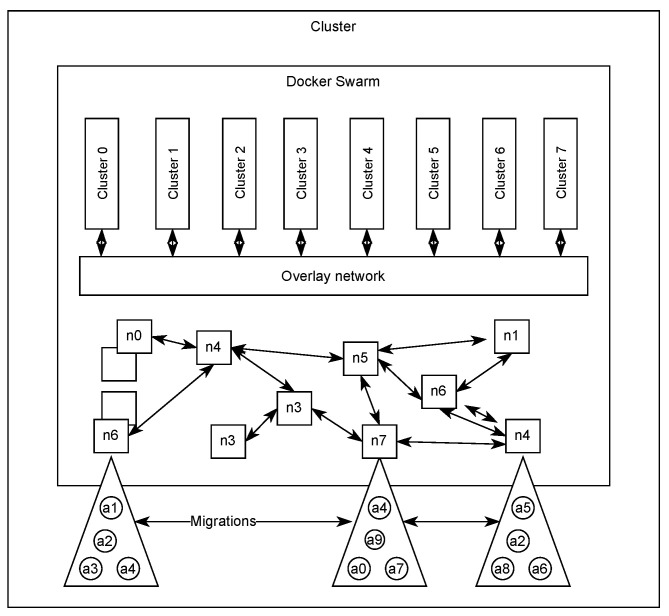
Cluster architecture.

Figure 6 outlines the architecture employed by the cluster. The swarm was created by defining an overlay network that could support more than the standard 256 IP addresses to accommodate our experiments. The complete reference implementation of the protocol, along with instructions, is available on the project’s Github at https://github.com/Nion-Network/Core accessed on: 30 April 2023.

A step-wise list of how the network is established using the protocol is given below:

1. The first node in the network is a bootstrapping node, and its public IP is known to all other nodes.
2. Each node is a Docker container that starts on a Docker swarm cluster.
3. A new node is spun up every 10 s to avoid unrealistic congestion.
4. Each node first connects to the bootstrapping node and joins the network.
5. Together, the bootstrapping node and all other nodes maintain a Kademlia-style DHT, which helps nodes discover each other.
6. The first block (genesis block) is created by the bootstrapping node only after a sufficient amount of validator nodes have joined the network.
7. To join the network as a validator, a node must send a signed inclusion request to everyone.
8. Initially, the bootstrapping node gathers inclusion requests, which will be included in the validator set once the genesis block is created.
9. The minimum number of nodes required to join the network is 1 + the size of the committee.
10. Once the first block is created, all nodes run a verifiable delay function and continue self-electing into roles and reaching consensus on each slot.
11. For every block, the block producer includes the digitally signed inclusion requests in the block so that other validating nodes who joined the network are considered as validators for the next slot.
12. The network continues to grow as new nodes join and send inclusion requests, and consensus is reached on each slot.
13. After all nodes are included in the validator set, an external service starts submitting containerized applications to the network via a POST request to the specific validator.
14. Validators receiving a request to run an application do so and start reporting the resources consumed.
15. Each following block contains a migration plan, which all nodes validate and execute in accordance with the protocol.

### 4.1. Consensus Layer

**Figure 7:**
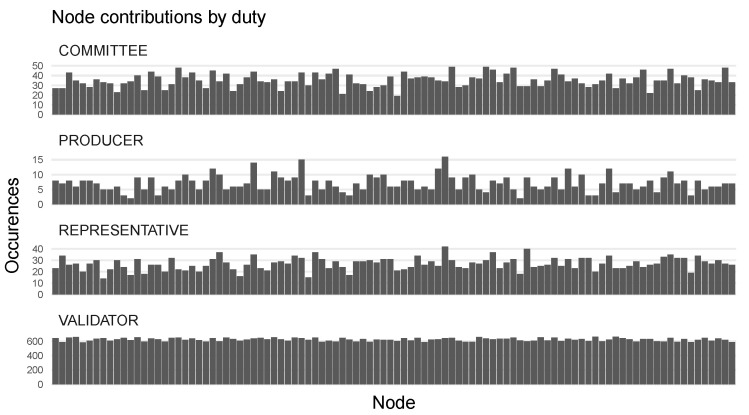
Distribution of roles across all participating nodes over a period of time: each bar chart shows the number of times a node has been elected to a specific role.

To verify that VDF-based consensus provides good decentralized randomness, we analyze the distribution of assigned roles. Figure 7 shows the frequency nodes were elected into individual roles. Additionally, since container resource consumption statistics are propagated through a decentralized k-means clustering, cluster representatives are also shown. We observe that nodes were elected into all roles, while there was some variance in the block producer role. This is due to the small sample size of 1000 slots in each, of which only one node is selected as the block producer. A more uniform distribution is expected with a larger sample size.

**Figure 8:**
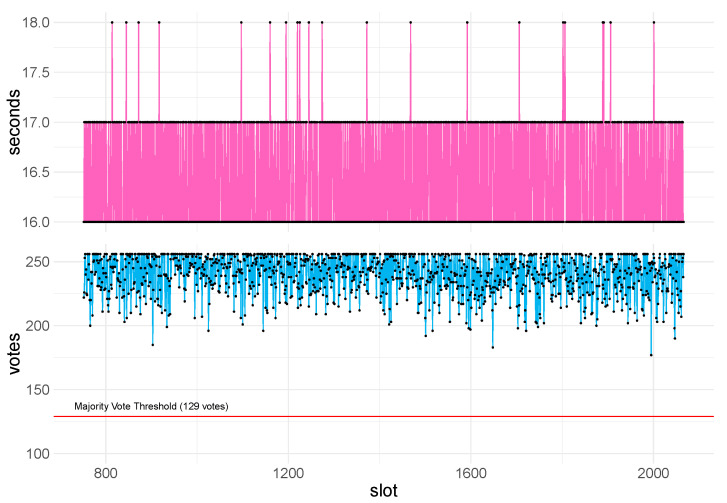
Committee vote aggregation and block times in a network of 1000 nodes and 256 committee members.

To validate the scalability of the consensus layer, we examined a network of 1000 nodes with a committee size of 256 nodes and a target block time of 16 s. Figure 8 shows the block times, and the number of votes per block that were successfully aggregated within the time window for the given slot. We observe that all proposed blocks were accepted as the majority vote threshold was surpassed, and no skip blocks were produced despite the low block time and size of the committee. Moreover, we observe almost no variance in block time, indicating that the system had no issue propagating messages.

### 4.2. Orchestration and Migration

**Figure 9:**
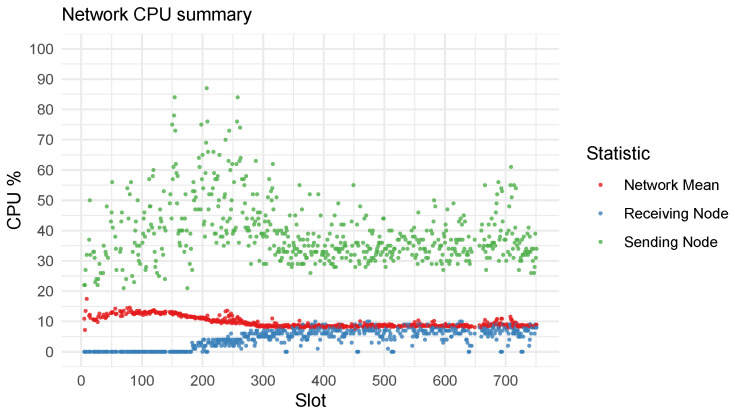
CPU load distribution of the entire network over the last 750 slots.

One of the most important features of the system is the ability to migrate applications in a decentralized, transparent, and verifiable way. The decentralized orchestrator aims to distribute load across the network evenly by migrating applications away from nodes with heavy load to those with resources available. To test the performance and efficiency of migrations, we consider the worst case scenario in which all applications were submitted to one node. Figure 9 illustrates the CPU load of nodes across the last 750 slots because nodes join the network gradually and affect the early distribution, which skews the observations. We observe that the orchestration algorithm migrates applications away from nodes with high CPU consumption to nodes with more available resources. This resulted in a gradual decline in the mean CPU load across the network.

**Figure 10:**
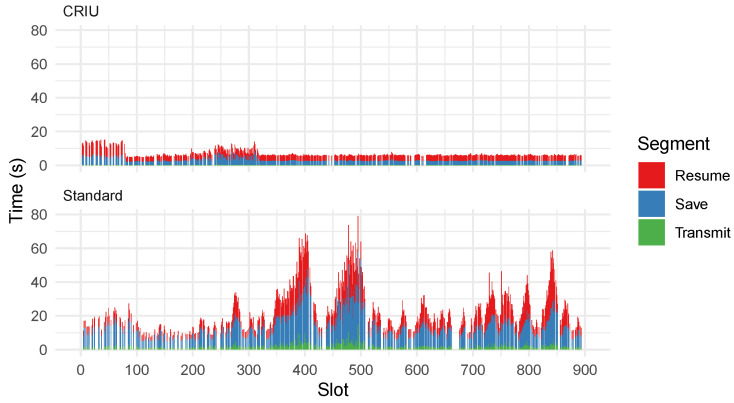
Migration timing comparison between standard and CRIU-enabled migrations.

In Figure 10, we compare migration times of both test-nets to evaluate the feasibility and performance of CRIU enabled migrations. We break down a migration into three steps: *Save*, *Transmit*, and *Resume*. For standard migrations, saving requires pausing the running container, exporting, and compressing it. In CRIU migrations, the container is paused but not exported. Only the state of the container is extracted and compressed. After transmission, the receiving node must resume the container. In standard migrations, the container is uncompressed and resumed. While using CRIU, a new container from the same base image is created, and the uncompressed state is injected into it.

Using CRIU, the payload for transmission is much smaller, and hence the transmission time is greatly improved over standard migrations. Additionally, compression is a CPU-intensive task. Compressing and decompressing only the state of an application instead of the entire container is considerably faster. The median uncompressed exported state of the application using standard migration was 142.2 MB. Using CRIU, the median size of the uncompressed state was 15.2 MB. The spikes in standard migrations can be attributed to a lack of resources, as nodes under heavy stress from running other applications lack the resources needed to perform the compression promptly. Table 3 provides a statistical summary of the observed times in milliseconds. We observe that CRIU-enabled migrations are not only faster but also produce more consistent migration times. This can be observed by the considerably lower standard deviation in Table 3.

**Figure 11:**
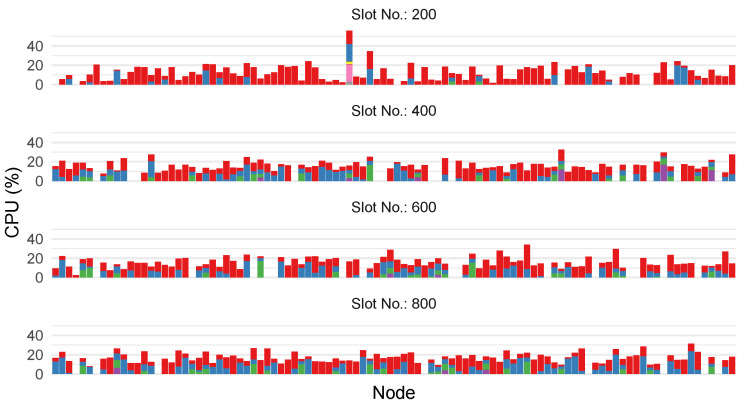
Discrete time visualization of applications and their CPU load utilization on participating nodes. Colors indicate individual containers active on each node and are not necessarily the same container on different nodes.

Another way to visualize the dynamics of the system is shown in Figure 11. Each bar chart shows nodes on the x-axis. Stacked bars are used to illustrate the number of applications running on the node and their respective CPU consumption in %. We observe that, initially, the application distribution was uneven, with a very high CPU load on one node. This is the result of submitting incoming applications to one node. Over time, the system is able to evenly distribute applications across the network.

### 4.3. Network Clustering

**Figure 12:**
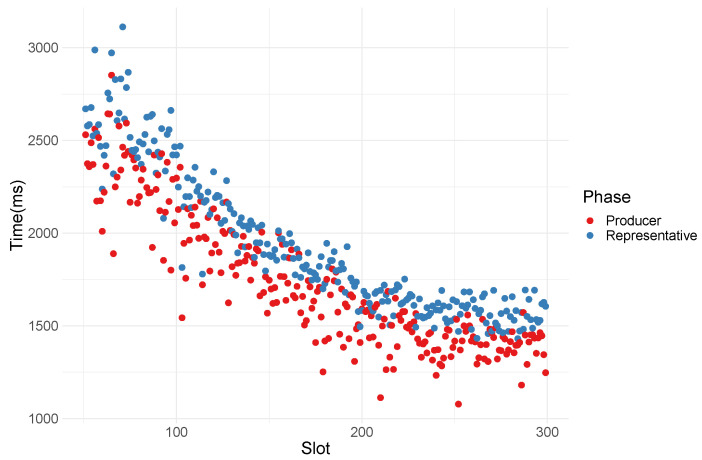
Time distribution of resource propagation in two phases. Initially, validators submit their resource statistics to their respective cluster representatives (*To representative*). After, cluster representatives send collected reports to the block producer (*To Producer*). There were a total of 50 clusters created each slot within a target slot time of 16 s, which sets the upper bound for resource propagation at 5.4 s.

The performance of the decentralized orchestrator heavily depends on the propagation speed of resource allocation from all validators. In a clustered network, *validators* report their resources to their cluster *representatives*, which then send an aggregated report to the block producer. To avoid a potential attack vector on the clustering, the network topology changes every slot. Figure 12 shows the time needed to deliver the resource reports to *representatives* and, finally, the *producer*. We observe that in the first few minutes, while the nodes are joining the network at a high frequency, the propagation times are slower (still well within $\frac{1}{block\_time}$) but stabilize quickly, even with networks of 1000 nodes.

## 5. Discussion

Our experiments demonstrate that the reference implementation of the protocol behaves as intended. We found that employing verifiable delay functions as a source of entropy for role election provided an effective means of ensuring security while achieving consensus. Throughout our extensive testing, we closely monitored telemetry data from individual nodes as well as the overall system, but did not observe any significant deviations from expected behavior, except during the initial phase when several nodes joined the network simultaneously. We consider a network size of 1000 nodes to be sufficiently large and representative for drawing conclusions, although we acknowledge that larger networks could be tested if hardware constraints were not a limiting factor.

It should be noted that the proposed protocol does not constitute an incremental contribution to existing autonomous container orchestration protocols, and thus a comparative analysis is not applicable. Overall, our observations indicate that the absence of unexpected behavior during testing is a positive indication that the protocol performed as expected based on its theoretical assumptions.

## 6. Conclusions

In this paper, we introduced a decentralized architecture capable of run-time application migration for large-scale deployments of peer-to-peer IoT sensor networks. We describe three key contributions: a scalable consensus protocol layer; an efficient, secure, and dynamic topology; and a decentralized orchestrator capable of low-latency real-time application migrations.

We evaluate each contribution by performing empirical tests with our reference implementation of the protocol. Additionally, we improve migration times by implementing CRIU, an experimental feature of Docker that allows the system to migrate an application’s state without affecting its run-time. Using CRIU-enabled migrations, we observe considerable reduction (nearly 10-fold) and improved consistency in migration times.

The results of our experiments show that distributed consensus and application management is possible at run-time, thus opening the door to several improvements towards self-managing IoT platforms. The increase in network usage and CPU load has been shown to be acceptable when taking into account the scalability, fault tolerance, transparency, and absence of an SPOF that our solution brings. More importantly, we have shown that blockchain overhead is a negligible aspect of the actual cost of application migration as the system is able to finalize blocks with slot times as low as 5 s while maintaining higher decentralization than existing platforms.

As future work, network instability (devices entering and leaving the network) should be explored, and solutions to reduce the required computational power while maintaining optimal application management should be investigated. Moreover, the algorithm governing the decentralized orchestrator will be extended to allow applications to submit migration policies that the orchestrator will have to follow during its operation. As future work, more efficient orchestration algorithms should be explored, with emphasis given on performing multiple migrations in the same slot with a non-cycling constraint.

Furthermore, geo-sharding the network must be explored. In a geo-sharded network, nodes participating are assigned into shards based on their geographical location. A weaker consensus within a shard can speed up the state transition by periodically snapshotting sharded states into the main chain. This will enable applications to specify more complex migration policies by limiting a geographical area within which the application may run (geo-fencing) or to improve network latency by migrating applications closer to clients of specific regions.

## Figures and Tables

**Table 1:** Table of Abbreviations.

Abbreviation	Long Name
VDF	Verifiable Delay Function
SPOF	Single Point Of Failure
UTXO	Unspent Transaction Output
PoW/PoS	Proof of Work/Proof of Stake
API	Application Programming Interface
CRIU	Checkpoint/Restore In Userspace
CPU	Central Processing Unit
RAM	Random Access Memory
PBFT	Practical Byzantine Fault Tolerance
RNG	Random Number Generator

**Table 2:** An example of a data block.

V	Node	RAM	DISK	CPU	Average Latency
$v_{0}$	A	50%	23%	90%	23 ms
$v_{1}$	B	47%	87%	23%	33 ms
$v_{2}$	C	12%	25%	15%	51 ms
$v_{3}$	A	35%	14%	56%	101 ms
$v_{4}$	D	25%	74%	16%	9 ms

**Table 3:** Summary of migration times in milliseconds.

Type	Segment	Min.	Max.	Med.	Mean	SD
CRIU	Resume	2366	10,012	3259	3540	1166
CRIU	Save	1975	8368	2701	3126	1111
CRIU	Transmit	46	833	79	88	61
Standard	Resume	1449	34,337	7414	9550	6637
Standard	Save	4010	49,080	11,231	12,875	6942
Standard	Transmit	506	15,007	1624	2047	1467

## Data Availability

Not applicable.
